# Prevalence of dementia and mild cognitive impairment among the older prisoner population in England and Wales: a cross-sectional study

**DOI:** 10.1136/bmjopen-2024-095577

**Published:** 2025-04-09

**Authors:** Katrina Forsyth, Baber Malik, Roger Webb, Leanne Heathcote, Laura Archer-Power, Richard Emsley, Jane Senior, Rachel Domone, Salman Karim, Adrian Hayes, Matthew J Carr, Alistair Burns, Jennifer Shaw

**Affiliations:** 1 The University of Manchester, Manchester, UK; 2 School of Community Based Medicine, The University of Manchester, Manchester, UK; 3 Health & Justice Research Network, The University of Manchester Faculty of Biology Medicine and Health, Manchester, UK; 4 Lancashire Police, Manchester, UK; 5 Department of Biostatistics and Health Informatics, Institute of Psychiatry, Psychology and Neuroscience, King’s College London, London, UK; 6 University of Manchester, Manchester, UK; 7 Lancashire Care NHS Foundation Trust, Preston, UK; 8 Somerset Partnership NHS Foundation Trust, Taunton, UK; 9 Department of Psychiatry and Behavioural Sciences, University of Manchester, Manchester, UK; 10 Offender Health Research Network, University of Manchester, Manchester, UK

**Keywords:** Dementia, Prevalence, Prisons

## Abstract

**Abstract:**

**Objectives:**

To estimate the prevalence of dementia and mild cognitive impairment (MCI) in the older prisoner population in England and Wales and to establish risk of harm to self and others, activity of daily living needs and social networks of prisoners with likely MCI and dementia.

**Design:**

We screened 869 older prisoners (aged 50 years and older) using the Montreal Cognitive Assessment (MoCA). Participants testing positive on the MoCA (≤23) were interviewed using the Addenbrooke’s Cognitive Examination, Third Revision (ACE-III) and a range of standardised assessments were used to assess risks of externalised violence and of self-harm; activities of daily living needs; mental health needs; history and symptoms of brain injury (if applicable) and social networks.

**Setting:**

The sample was drawn randomly from women’s prisons (n=10) and a representative range of adult men’s prisons (n=11) across England and Wales.

**Participants:**

Participants were aged 50 or over and resident in one of the participating prison establishments on the study’s census day.

**Main outcome measure:**

ACE-III.

**Results:**

We recruited 596 men and 273 women prisoners. Across the whole sample of older prisoners, the prevalence of dementia was 7.0% (95% CI 5.5%, 8.9%) (when weighted for sex and age), with the highest prevalence found among prisoners aged 70 years and older at 11.8% (95% CI 8.0%, 17.1%). The prevalence of dementia for men was 7.0% (95% CI 5.2%, 9.4%) and for women was 6.0% (95% CI 3.8%, 9.5%). Only two individuals (3%) who screened positively on the MoCA had a diagnosis of dementia in their prison healthcare notes, suggesting current under-recognition. The prevalence of MCI was 0.8% (95% CI 0.4% to 1.7%, weighted by age). Of those who screened positively on the MoCA, 32 (46%) participants had a high or very high risk of harm to self or others, and 70 (35%) had no friends with whom they could talk to about private matters or to call on for help (n=35, 50%).

**Conclusions:**

Approximately 1020 older adults living in prison have symptoms of likely dementia, and service provision for this group is inadequate.

**STRENGTHS AND LIMITATIONS OF THIS STUDY**

- This study is the first to estimate the prevalence of dementia and mild cognitive impairment (MCI) among men and women prisoners in England and Wales.
- These estimates are, however, based on the Addenbrooke’s Cognitive Examination, Third Revision and not a clinical diagnosis by a mental health professional.
- We were unable to distinguish the prevalence of subcategories of dementia.
- We were also unable to distinguish between a likely diagnosis of dementia or MCI and other conditions presenting with cognitive impairment, including learning disability, severe depression or hearing impairment.
- For the Montreal Cognitive Assessment, the suggested cut-off score of 23 should be further explored.

## Introduction

 Dementia is currently a National Health Service (NHS) priority.^1^ There are approximately 850 000 people with a diagnosis of dementia in England and Wales, with the prevalence projected to double by 2040.^2^ Recent research has focused on the suitability or otherwise of prison environments for individuals with dementia.^3^ Systematic reviews examining dementia in prison have emphasised the need to identify prevalence and improve services in line with community provision.^4^,^6^

In June 2023, there were 85 851 people in prison in England and Wales; 17% of the total prison population were older prisoners, defined as aged 50 or over.^7^ In England and Wales, prisoners aged 60 and older are the fastest growing group, followed by those aged 50–60.^7^ A similar pattern has been shown in other high-income countries, including the USA,^8^ Australia,^9^ Japan^10^ and Canada.^11^ This trend is likely to continue.^12^ This growth is in part due to an overall ageing population but also to increases in sentence length and the rise in frequency of retrospective historical sexual offence convictions.^13^

Dementia among prisoners is understudied and research is yet to establish what care should be provided for prisoners with dementia or mild cognitive impairment (MCI). In England and Wales, research so far has excluded women, likely due to the low number of women in prison aged 50 and over,^3^ and limited with respect to geographical and prison type representation.^14 15^ Dementia prevalence of 1%–13% among older male prisoners has been reported from previous studies in England and Wales.^14 15^ These estimates were generated from small samples that did not include women and may thus not be representative of the whole older prison population. Prevalence estimates also varied due to discrepancies in assessment measures used. A previous study from our research group^15^ investigated a sample of older, male prisoners drawn from a 1-day census in 12 prisons across the North West of England. They reported that 7% of participants achieved a high score on the Mini Mental State Examination,^5^ indicating possible dementia. Globally, the prevalence estimates for dementia in England and Wales have been as high as 44% of the older prisoner population.^16^

Studies have shown that prisoners with dementia more frequently experience multiple adverse consequences in relation to victimisation and punishment for non-adherence to prison rules.^4^,^6^ Furthermore, the prison environment in its current form is unable to effectively provide care for prisoners with dementia or MCI. Excessive noise and poor lighting can be distressing and disorientating to affected individuals^17^; often prisons are dark and use inadequate artificial lighting. Likewise, prison regimes are restrictive and, if disoriented, an affected prisoner may find it increasingly difficult to follow routine.^4^,^6^ There is also a lack of access to specialised services for older prisoners with suspected dementia or MCI^6 17 18^ and a need to develop/validate appropriate screening tools for prison.^16^ It is, therefore, imperative to estimate prevalence accurately and precisely to inform our understanding of the ability of current services to identify and manage prisoners with dementia or MCI and to establish what care should be delivered and what adjustments should be made to the prison environment.

This study aimed to:

1. Estimate the current prevalence of dementia and MCI in the older prison population in England and Wales.
2. Establish the risk of harm to self and others, activities of daily living needs and social networks of those who screen positive on the Montreal Cognitive Assessment (MoCA).

## Methods

We aimed to recruit 860 prisoners (591 men and 269 women). This sample size was calculated to enable estimation of a prevalence value of 7%, based on a previously reported estimate,^14^ with 2% precision (95% CIs 5%, 9%, applying sex-specific finite sample corrections).

### Eligibility criteria

Participating prisoners were aged 50 or over and resident in one of the participating prison establishments on the study’s census day. The following exclusion criteria were applied: (1) Considered by prison or healthcare staff as being unsafe to interview alone due to their current risk; (2) Did not have a functional command of the English language; (3) Lacked capacity to provide informed consent and an appropriate personal or independent consultee could not be identified/contacted or (4) unwilling to be consulted or made the decision to refuse consent.

### Informed consent

A research team member explained the project to the eligible prisoners and gave them the information sheet as well as explaining their rights. Researchers received training in assessing capacity in accordance with the Mental Capacity Act (MCA) 2005. Researchers also sought an opinion from prison healthcare staff regarding capacity and, if it was deemed that a prisoner lacked capacity, an attempt was made to identify a ‘personal consultee’ as defined by the MCA to advise on the individual’s participation. In the first instance, even when participants were considered to lack the capacity to consent to participation, researchers asked if they could contact someone else to advise on the individual’s behalf. Potential consultees from outside of the prison were only contacted if the research team established that they were aware that the potential participant was in prison and that they had difficulties that limited their capacity to consent. The initial approach to anyone outside of the prison was made by prison healthcare staff.

### Sampling

The sample was drawn randomly from all women’s prisons (n=10) (with the exception of one Women’s prison that refused to participate) and a representative range of adult male prisons (n=11) across England and Wales, including local prisons holding individuals on remand, serving short sentences and in the early part of long sentences; training and dispersal prisons holding men part-way through long sentences; high-secure establishments holding those considered to be high risk; and open prisons holding short-term prisoners deemed low risk and those in the final stages of long sentences in preparation for community release. We selected sites based on the proportion of each prison type in the prison estate as a whole—1 of the 8 high secure sites; 3 of the 31 local prisons; 5 of 51 category B or C training and dispersal prisons and 1 of the 7 category D/open prisons (as defined by the justice.gov.uk prison index). We selected the prisons from across England to include a broad geographical spread and for pragmatic reasons. This included one prison in the North East, three in the North West, two in Yorkshire, one in the midlands and two in London. We also included a prison that has a specific wing for older and disabled prisoners. This was one of only two prisons in the country with a dedicated wing for prisoners based on their age and health or social care needs. One of these two prisons was selected for pragmatic reasons. Once the potential prisons had been identified, we wrote to the governors to request access.

We established the total number of prisoners aged 50 and older in the recruiting prisons (Table 1) and used this information to calculate a sampling fraction, which determined the proportion of prisoners to be approached at each site. We stratified our sample according to age, an important variable when investigating dementia and MCI, as prevalence doubles with every increase of 5 years.^19^ Within each site, we sampled 30% of those aged 50–59; 60% of those aged 60–69, 90% of those aged 70–79 and all prisoners aged 80 or over.

**Table 1:** Number of men and women in prison in England and Wales by age

Age group	All	Men	Women
50–59	8638	8227	411
60–69	3243	3149	94
70+	1641	1624	17
Total	84 373	80 454	3919

### Recruitment and data collection procedures

We estimated point prevalence according to a specific census date per site. A research nurse or other appropriate staff member within each of the prisons acted as a single point of contact. The research team provided the contact at each site with a census date, and this individual then identified potential participants who met all of the inclusion criteria. Once a numbered list of all potential participants had been generated, the contact informed researchers of the number of eligible older prisoners identified. A random number generator was used to create a list of potential participants. An information and appointment slip was sent to each of the individuals who had been randomly selected as being potential participants. If individuals did not attend their appointment, the single point of contact went to visit the individual to check that they had not missed their appointment unintentionally.

If an individual consented to participating, the researcher conducted an initial interview, which included the collection of demographic data and completion of the MoCA. For individuals who scored positively on the MoCA, they proceeded to a further needs assessment interview. During the second interview, researchers first obtained additional, more detailed demographic information. Information on current physical and mental health as well as any diagnosis of learning difficulties or disabilities, sensory difficulties and/or use of substances/medication was gathered. This was supplemented by routinely collected information gathered from SystmOne electronic health records (EHRs). SystmOne provides a single EHR for every patient. This shared record is available across all healthcare settings to any staff who need it during a patient’s care. Participants consented to researchers accessing their EHR at each site. Researchers used a specifically designed proforma to collect information on past and current comorbidities and medications from the EHRs. Researchers interviewed participants using a range of standardised assessments to assess ADL needs; mental health needs; brain injury and social networks. For those individuals who gave their informed consent, risk and follow-up data were also collected at this stage. Information pertaining to risks of self-harm and reoffending was sought from the Offender Management Unit. Consent was sought from the participant (or advice from the consultee) as regards researchers accessing prisoners’ EHRs, which were subsequently screened for any indication of diagnosed MCI or dementia.

### Measures

All participants were invited to complete the MoCA^20^ between November 2016 and February 2018. A demographic proforma was designed for the study covering: health (including self-reported ADL problems, eyesight and hearing problems, learning difficulties, head injuries, whether the individual was under the influence of any substances); criminal justice information; and English language fluency. An additional bespoke proforma was completed to ascertain past and current comorbidities and medications. This information was derived from prison EHRs.

Participants who scored less than 23 on the MoCA were invited to complete a further battery of assessments. This was conducted as soon as possible after the initial assessment but not on the same day. The Addenbrooke’s Cognitive Examination–Third Revision (ACE-III^21^) was used to further assess cognitive function. Neither the MoCA nor ACE-III has been validated for use in prison.

The Bristol Activities of Daily Living Scale (adapted version^20^) was used. Twenty items were reduced to 18 for the purposes of this study, as questions relating to activities that are not relevant in prison (use of public transport; managing finances) were removed, while those referring to shopping and housework were rephrased to relate to canteen ordering and keeping one’s cell area clean. As a result, we did not look at the overall score but at individual items of the assessment. The Geriatric Depression Scale-15 was used to identify depressive symptoms.^21^ PrisnQuest^22^ was used to screen for mental illness in prison. The Rivermead Post-Concussion Symptoms Questionnaire^23^ was completed by those who reported a previous brain injury. The Lubben Scale-18 (modified version^24^) was used to measure social networks. These tools have previously been used in prison-based studies^15 25^ but have not been specifically validated for the prison population. We also sought the following information from the OMU in each prison: (1) Offender Assessment System (OASys) rating of risk of harm to self and others (This incorporates static and dynamic risk and is categorised as very high, high, medium or low. It is reassessed at different time points depending on the individuals’ sentence type and prior to release). (2) Risk Matrix 2000 score (a risk assessment tool which uses static risk factors to predict the likelihood of reconviction for a sexual or violent offence). The information from the OMU and EHRs was collected after all assessments were complete.

### Data analysis

The prevalence estimates were directly age-standardised using the age distribution of the national prison population as the reference. From aged 50 years and older, the following age strata were applied: 50–59, 60–69, 70+. Each stratum-specific weight was calculated as the proportion of the whole prison population that was in that age group divided by the equivalent proportion for the study sample. We then calculated the adjusted numerator by taking the product of the weight and the study sample size within each age stratum. We produced age-specific prevalence estimates to enable comparison with community-based samples.^1^ Missing data were minimal and, therefore, it was not necessary to compute any missing data.

### Patient and public involvement

A postdiagnostic dementia support group, based at Greater Manchester Mental Health NHS

Foundation Trust, assisted in the development of information sheets, consent forms, etc. This helped to ensure that these documents were formatted appropriately.

An expert by experience sat on the Study Steering Committee, was involved in the management of this research study and provided his expertise responsively throughout the life of the project.

## Results

### Prevalence

**Figure 1:**
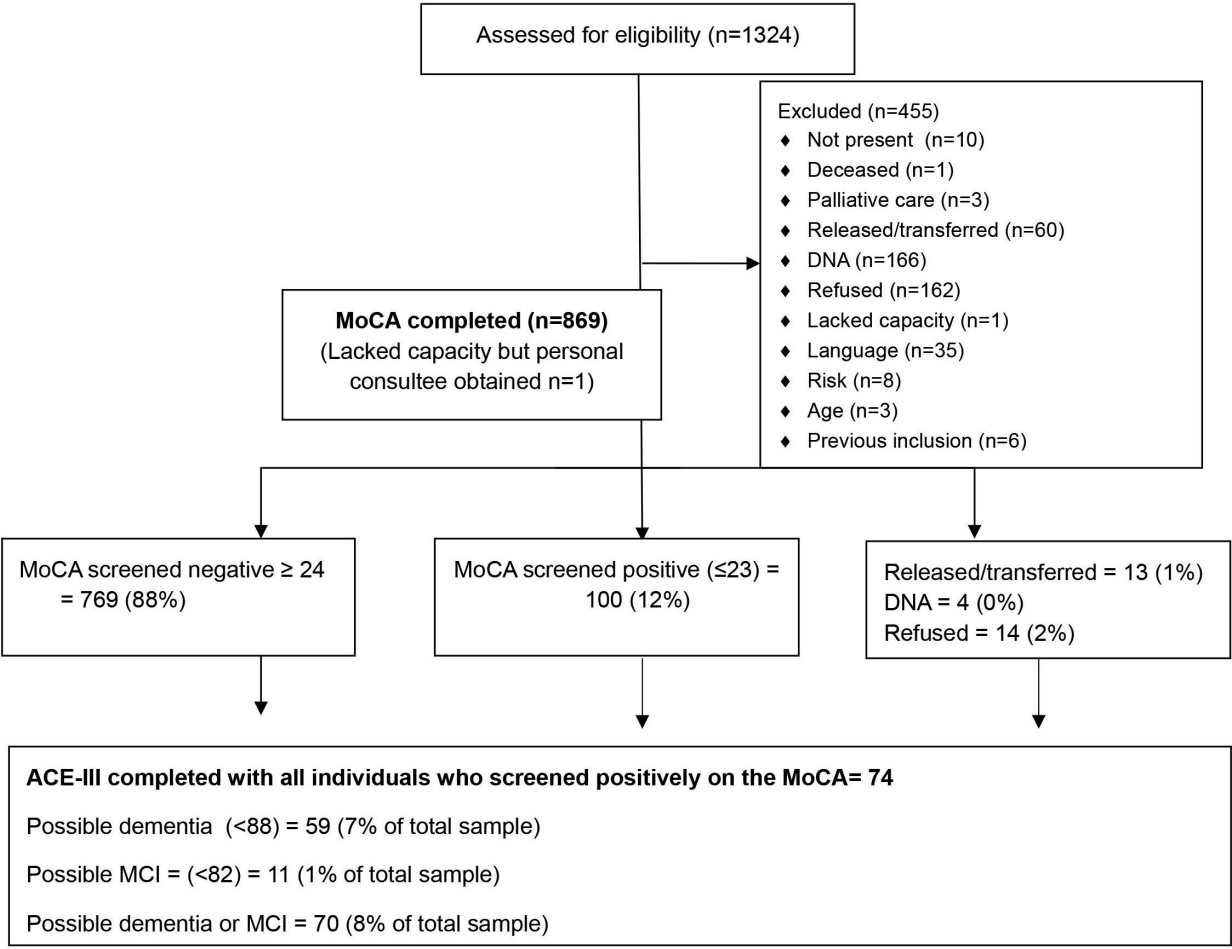
Dementia and mild cognitive impairment prevalence estimation flow diagram. ACE-III, Addenbrookes Cognitive Examination, Third Revision; MCI, mild cognitive impairment; MoCA, Montreal Cognitive Assessment.

We recruited 869 prisoners; 596 men and 273 women. The details of participants’ recruitment are given in figure 1.

As shown in figure 1, 100 participants screened positive on the MoCA (12%), and 70 (8%) of the total sample screened positive on the ACE-III. This included 11 individuals who screened positive for possible MCI (1% of the total sample) and 59 who screened positive for dementia (7% of the total sample).

Across the whole sample of older prisoners, the estimated prevalence of dementia was 7.0% (95% CI 5.5%, 8.9%) (when weighted for sex and age), with the highest prevalence found among prisoners aged 70+11.0% (95% CI 7.4%, 16.2%). Participants aged 50–59 had the second highest estimated prevalence at 6.1% (95% CI 4.2%, 8.8%), followed by prisoners aged 60–69 (5.0%; 95% CI 2.9%, 8.5%). We therefore, estimate that there are 1020 older prisoners with symptoms of dementia in England and Wales. Only two individuals (3%) who screened positively on the MoCA had a diagnosis of dementia in their prison healthcare notes. This suggests current underdetection when compared with the findings of our assessments.

There are 14 578 prisoners aged 50 years and older in England and Wales^9^ (June 2023). It is estimated that 7% of these prisoners have symptoms of dementia. This equates to an estimated 1020 older prisoners with dementia symptoms in England and Wales. It should be noted that these findings are based on validated cognitive impairment assessments and not a clinical diagnosis. The prevalence of dementia for men was 7.0% (95% CI 5.2%, 9.4%) and for women was 6.0% (95% CI 3.8%, 9.5%).

### Characteristics of participants screening positive on the MoCA

70 (95%) participants were screen positive on the MoCA and also went on to screen positively on the ACE-III (for MCI or dementia). Demographic information concerning these participants is detailed in table 2 (online supplemental appendix). Most prisoners were White British (55, 79%), and 25 (36%) of the sample were employed prior to becoming prisoners. The mean number of previous convictions was 2.95 and the mean current sentence length was 18 months.

**Table 2:** Prevalence estimates of dementia in each age group (standardised by gender)

Age group	NumberSampled	Dementia cases	Prevalence(95% CI)
50–59	425	26	6.1 (4.2, 8.8)
60–69	249	10	5.0 (2.9, 8.5)
70+	195	23	11.0 (7.4, 16.2)

### Other conditions potentially causing MCI or dementia

There are several possible reasons for cognitive impairment in this sample other than dementia/MCI, including severe depression, stroke, chronic alcohol misuse, serious head injuries, other neurological conditions and learning disability. Hearing impairment may also have affected performance on the ACE-III. One participant had hearing impairment entered in their EHRs. However, 23 individuals reported some hearing problems at interview. Three participants had a learning difficulty in their EHRs; however, 21 self-reported a learning disability, including 10 who attended a Special Educational Needs school. One person had a brain injury in their EHR with a further 22 prisoners self-reporting a history of brain injury.

We also examined potential comorbidity. 42 participants screened positively on the Geriatric Depression Scale short version (60%), indicating a need for further clinical exploration. Only 13 of these individuals had a diagnosis of depression entered in their prison EHR. Seven participants (10%) who screened positive on the MoCA scored 3 or above on Prisonquest, indicating that further clinical assessment for mental illness was required. 19 (27%) reported a history of head injury on the Rivermead Post Concussion Questionnaire, and 9 (13% of ACE-positive people) reported experiencing poor memory after the head injury.

19 (27%) of participants screened positive on the ACE-III for ADL dependence. The domain with the highest number of participants who experienced difficulties was mobility (n=21, 30%). 32 (46%) participants had a high or very high risk of harm to self or others as measured by the OASys. In addition, four (6%) participants had obtained high/very high scores on the RM2000, indicating elevated sexual offending risk. 70 (35%) participants who screened positively on the ACE-III had no friends to talk to about private matters or to call on for help (n=35, 50%). In addition, over half of these participants (37, 53%) stated that they ‘never’ had a friend to talk to when they had an important decision to make.

### Comparisons to the community

We compared the prevalence rates of dementia among the older prisoner population with those that have been reported for the wider community.^1^ Data were unavailable for individuals aged 50–59 living in the community. Community prevalence estimates are broken into 5-year age brackets; we, however, used 10-year age groups. It is estimated that 0.9% of individuals aged 60–64 and 1.7% of individuals aged 65%–69% living in the community have dementia.^26^ The dementia prevalence rate among our sample of older prisoners is approximately 3–4 times (5%) higher for individuals aged 60–69 than it is for those living in the community.

The median age of individuals aged 70+ in our sample of older prisoners was 73 years old. The prevalence of dementia in those aged 70–75 living in the community is 3%.^26^ Consequently, we can estimate that older prisoners aged 70 years and older are 3.5 times more likely to have a diagnosis of dementia than their age-matched counterparts living in the community.

## Discussion

### Main findings

Across the whole sample of older prisoners, the prevalence of dementia was 7.0% with the highest prevalence found among prisoners aged 70+. Participants aged 50–59 had the second highest estimated prevalence at 7.0%, followed by prisoners aged 60–69. Only two individuals (3%) who screened positively on the ACE-III had a diagnosis of dementia in their prison EHR, suggesting current under-recognition. These figures may be accounted for in part by higher rates of alcohol misuse (and associated Korsakoff psychosis) in prisoners.^27^ It is also possible that the higher prevalence in the prison population of brain injury and vascular disease (associated with vascular dementia) could contribute to these prevalence figures.^28^

### Comparisons with findings from previous studies

This study is the first to have comprehensively examined the prevalence of dementia and MCI in the population of older prisoners in England and Wales using a sample that is representative in terms of the gender breakdown of older prisons, geographical areas, prison types and that entailed a rigorous two-stage assessment process. It is also the first prevalence study conducted on this topic to weight findings according to sex and age distributions. Our result of 7.0% is higher than some previous results^14 29^ and lower than others,^29^,^31^ with previous estimates ranging between 1% and 20% globally.

Our estimated prevalence figures were considerably higher than the numbers estimated by healthcare managers and prison governors in a national survey distributed by the research team,^31^ suggesting significant under-recognition of dementia/MCI in prisons and potentially prior to prison. Some of this may be explained by variance in how dementia was measured (eg, clinical diagnosis, screening tools). However, estimated prevalence rates globally are as high as 44%.^16^

### Strengths and limitations

This study is the first to estimate the prevalence of dementia and MCI among men and women prisoners in England and Wales. We established a representative sample of the national prison population and had sufficient power to estimate the prevalence of dementia and MCI. These estimates are, however, based on the ACE-III and not a clinical diagnosis by a mental health professional. Therefore, our prevalence figures may represent an overestimation. Furthermore, we were unable to distinguish the prevalence of subcategories of dementia. We were also unable to distinguish between a likely diagnosis of dementia or MCI and other conditions presenting with cognitive impairment, including learning disability, severe depression or hearing impairment. We know, however, the proportion of people screening in on the ACE-III who had these conditions. However, we do not know whether they had both conditions, dementia and a learning disability, or just one of them. It is possible that this led to an overestimation of the prevalence of dementia and MCI in our sample.

We used several measures to characterise people who screened positive on the ACE-III, some of which had limitations. Most had not been used previously in prisoner populations, and it is likely that cut-off scores require adjustment for this population. Risk was estimated using the OASys to indicate the person’s risk of reoffending. Risk data are important when service provision is considered, and it is questionable whether this kind of risk data is adequate when considering release or placement in a secure nursing home. It is possible that a more appropriate risk assessment tool would need to be found, adapted or developed for prisoners with MCI/dementia.

For the MoCA, we used a cut-off score of 23 as screening positive warranting further assessment. This was adopted because several studies question whether or not the universal cut-off score for the MoCA of 26 points (developed originally on 90 Canadian healthy control individuals) was appropriate across disadvantaged populations with low literacy and education levels, and for individuals whose first language is not English.^32 33^ There are, however, limitations in using screening tools designed for use in the community rather than prison.

Overall, we estimated that 1020 people across the prison estate in England and Wales experience symptoms of dementia. We aimed to estimate the likely change in number of people in prison with MCI/dementia in future years. We know that there has been a significant rise in the population of people over the age of 50 in recent years, largely due to an increase in sentencing for historical, mainly sexual offences.^13^ Unfortunately, we were unable to estimate with any certainty whether this rise is likely to continue. This is largely because we were unable to ascertain whether this sentencing practice is likely to proceed at the same rate or whether sentencing for these historical offences has now plateaued.

### Future research

It would be useful to conduct a cohort study to establish the health, social care and criminological outcomes of a sample of MoCA and ACE-III-positive individuals over a period of 3–5 years. Future research should establish accessible pathways of care for individuals with suspected dementia or MCI in prison. It would also be helpful to validate the MoCA for use in prison populations in future studies.

### Conclusions

In summary, we found that the prevalence of dementia was 7.0% for ages 50+ and 11.8% in prisoners 70+. In addition, the majority of prisoners who screened positive on the ACE-III had a high or very high risk of harm to self or others and reported having no friends to call on for help. There is a lack of care pathways, specialised services and equivalent services to those provided to those living in the community. The continued increase in the number of older prisoners means care provision for this disadvantaged group needs addressing.

## Supplementary material

10.1136/bmjopen-2024-095577 online supplemental file 1

## Data Availability

Data are available on reasonable request from the contact author.
