# Supplementary material for: Prevalence of dementia and mild cognitive impairment among the older prisoner population in England and Wales: a cross-sectional study
Source: BMJ Open. 2025 Apr 9;15(4):e095577. doi: 10.1136/bmjopen-2024-095577 (PMC11987132; doi:10.1136/bmjopen-2024-095577)
Supplement: online supplemental file 1 [file bmjopen-15-4-s001.pdf]

Appendix

Table 1: Demographic information about participants

**Ethnicity**	Frequency (% out of 70)
White British	55 (79%)
White Irish	4 (6%)
Black African/ Caribbean	4 (6%)
Asian/Pakistani	1 (1%)
Other	1 (1%)
**Marital Status**	
Single	22 (31%)
Separated/Divorced	8 (11%)
Married/Partner	33 (47%)
Widowed	7 (10%)
**Employment status**	
Full/part time employment	25 (36%)
Unemployed	13 (19%)
Retired	19 (27%)
Long term sick (Benefits)	12 (17%)
**Accommodation**	
Private	60 (86%)
House	
Temporary accommodation	2 (3%)
Homeless	2 (3%)
Supervised Hostel	3 (4%)
**Living Circumstances**	
Alone	33 (47%)
With Spouse/	13 (19%)
Partner with children	
With Parents	1 (1%)
With Spouse/Partner without children	16 (23%)
