# Supplementary material for: Prevalence of dementia and mild cognitive impairment among the older prisoner population in England and Wales: a cross-sectional study
Source: BMJ Open. 2025 Apr 9;15(4):e095577. doi: 10.1136/bmjopen-2024-095577 (PMC11987132; doi:10.1136/bmjopen-2024-095577)
Supplement: Supplementary file 2 [file bmjopen-2024-095577.reviewer_comments.pdf]

PEER REVIEW HISTORY

BMJ Open publishes all reviews undertaken for accepted manuscripts. Reviewers are asked to complete a checklist review form and are provided with free text boxes to elaborate on their assessment. These free text comments are reproduced below.

ARTICLE DETAILS

Title (Provisional)

Prevalence of dementia and mild cognitive impairment amongst the older prisoner population in England and Wales: a cross sectional study

Authors

Forsyth, Katrina; Malik, Baber; Webb, Roger; Heathcote, Leanne; Archer-Power, Laura; Emsley, Richard; Senior, Jane; Domone, Rachel; Karim, Salman; Hayes, Adrian; Carr, M; Burns, Alistair; Shaw, Jennifer

---

VERSION 1 - REVIEW

**Reviewer**	**1**
**Name**	**Prof. Margaret Chandlee Miller**
**Affiliation**	**University of South Carolina**
**Date**	**20-Dec-2023**
**COI**	**none**

Comments to the Author:

Thank you for the opportunity to review this paper. This is a fascinating topic and I thoroughly enjoyed reviewing it. The authors' manuscript makes an important contribution to the literature surrounding prison populations living with MCI or dementia. The manuscript provides information about the assessment of 869 prisoners 50 years and older. The researchers screened these individuals with the MoCA, ACE-III, and other standardized assessments. The prevalence of dementia was 7.0% for ages 50+ and 11.8% in prisoners 70+. In addition, the majority of prisoners who screened positive on the ACE-III had a high or very high risk of harm to self or others and reported having no friends to call on for help. This is a novel topic and this article has the potential to serve as a citable reference for many future manuscripts and provide evidence for the need of tailored treatment and care for individuals in the prison system with dementia. I would recommend some minor revisions, which mainly include providing additional information/clarification within the

text.

The areas that need to be addressed are as follows:

p. 7, line 6:
-Please spell out NHS.

p. 8, line 24: “We aimed to recruit 860 prisoners (591 men and 269 women). This sample size was calculated to enable estimation of a prevalence value of 7%, based on a previously reported estimate (14), with …”

-Why limit your ability to estimate a higher prevalence? Please expand on why this decision was made and why you did not aim to recruit more prisoners in your sample.

pg. 8, line 55: “by the MCA…”
-Spell out MCA or add MCA in paratheses after Mental Capacity Act in the previous text.

p 10, line 13 and 14, “This was supplemented by routinely collected information gathered from SystmOne electronic health records (EHRs).….”
-Please provide more detail about the EHR’s, the data they contain, and how they were used to supplement the information collected in the study. Was there a specific protocol in place or a standard set of variables that were pulled for each participant? What information was accessed? Who accessed this information? Were special permissions obtained? Clarification on this is needed.

p 10, line 20-21, “Information pertaining to risks of self-harm and re-offending was sought from the Offender Management Unit.….”
-Please clarify what self-harm and re-offending assessment information is included in the Offender Management Unit. When was this assessed? Is it reassessed periodically? Does it change during the individual’s sentence?

p 10, line 22-26, “Consent was sought from the participant (or advice from the consultee) as regards researchers accessing prisoners EHRs, which were subsequently screened for any indication of diagnosed MCI or dementia.”
-How many provided consent and how many did not? Could you have missed additional diagnoses?

p 10, lines 33-36, “The MoCA was assessed between November 2016 and February 2018 …. Participants who scored less than 23 on the MoCA were invited to complete a further battery of assessments.”
-Please clarify when further assessments were taken. It’s not clear if they were

performed Immediately after the MoCA or at another time. Also, when were the prisoner's EHR's accessed. A clear description of the timeline will be helpful.

p 11, line 29-32, "Patient and Public Involvement"
-Can this section be moved to the acknowledgements? It might fit better there.

p 12, Figure 1,
- Is it possible to report a comparison of those included in the study and those excluded to assess any potential bias?

p 13, line 18-20, "Only 2 individuals (3%) who screened positively on the ACE-III had a diagnosis of dementia in their prison healthcare notes, suggesting current under-detection. …."
-This doesn't agree with the conclusion on p16, line 22-24. Please review and clarify your take home message.

p 15, line 12-24, "We compared the prevalence rates of dementia amongst the older prisoner population with
those that have been reported for the wider community."
- Where did the "wider community" data come from? This information needs to be included in the methods section and ordered in line with how the results are presented.

p 15, line 41-43, "Our result of 7.0% is higher than some previous results 1-7%."
- These results are lower than similar studies in the US. See Miller, et al., Dementia in the incarcerated population: a retrospective study using the South Carolina Alzheimer's disease registry, USA. DOI 10.1108/IJPH-08-2021-0071. Also, take a look at other prevalence studies in Europe including Fazel et al., 2001; Kingston et al., 2011; Combalbert et al., 2018.

p 15, line 48, "…team (32), suggesting significant under-recognition of dementia/MCI in prisons."
- Also, potential missed dementia/MCI diagnosis prior to prison sentence.

p 15, line 51-56, "However, these differences may be actual and accounted for in part…."
- This paragraph has more to do with who was reporting the diagnosis and not why the prison population has high diagnosis. These lines would fit better on page 14, paragraph 1.

p 16, line 22-24, "Overall, it is likely that this led to an overestimation of the prevalence…"
- Need to cite some statistics comparing the number of diagnoses made by

physicians and those made by non-physicians using validated screening tools only. This is needed to confirm your conclusion here.

p 16, line 41-48, “For the MoCA, we suggest a cut-off score of 23…”
- Please expand on the implications of this choice. Has the MoCA been validated in similar populations? Be sure to include information on the need for studies to validate the use of MoCA in the prison population.

p 16, line 58, “…sentencing for historical, mainly sexual offences.”
- Is there any literature to support this? Please cite a reference(s) here.

p 17, line 9-15, “….outcomes of a sample of ACE-III-positive individuals over a period of 3–5 years”
- What about MoCA?

p 16, line 22-23, “In summary, we have found that 7% of the older prisoner population (1020 individuals) have dementia…”
- I suggest a revision like this to include all main findings:
"We found that the prevalence of dementia was 7.0% for ages 50+ and 11.8% in prisoners 70+. In addition, the majority of prisoners who screened positive on the ACE-III had a high or very high risk of harm to self or others and reported having no friends to call on for help."

Additional suggestions:
Please be sure all results are introduced in the discussion. I believe these will strengthen your paper and help its readability.

**Reviewer**	**2**
**Name**	**Stoliker, Bryce E.**
**Affiliation**	**University of Saskatchewan, Centre for Forensic Behavioural Science and Justice Studies**
**Date**	**04-Jan-2024**
**COI**	**none**

Comments to the Author:

This study assesses the prevalence of dementia and mild cognitive impairment among a nationally representative sample of 869 older people in custody to improve knowledge on the extent of this issue in the prison system in England and Wales. The authors used the ACE-III and MoCA to assess the sample. Overall, this study was well done and presents interesting findings, which contributes nicely to the growing body of work in this area. Below I have detailed specific comments/questions for the authors to address, which are relatively minor in nature.

General Comments:
- The authors should be cognizant of language throughout when it comes to reference of clinically significant symptoms of dementia (and cognitive impairment) vs. a dementia diagnosis. In this case, screening instruments are used and, thus, a formal diagnosis is not made. On pg. 13, line 36-38 the authors state, “It is estimated that 7% of these prisoners have dementia.” However, it is most appropriate to suggest that 7% of people in custody have clinically significant symptoms of dementia (and/or mild cognitive impairment).

ABSTRACT
- With respect to the “Setting” component of the Abstract, the authors should specify the n’s for prisons included in the study (separately for men and women prisons).

- With respect to the “Main Outcome Measure” component of the Abstract, is the MoCA not included as a main outcome?

- With respect to the “Results” component of the Abstract, a few minor points: (1) the authors should include prevalence estimates not only as a whole, but also for men and women separately regarding the ACE-III (dementia) and MoCA (MCI); (2) when referencing MCI it should be indicated in one way or another this is in reflection of MoCA screening results; and (3) findings for behavioural risk (i.e., harm to self/others) and needs/social networks are noted within the context of screening results for dementia (ACE-III), but what about mild cognitive impairment (MoCA)? An additional minor point: Pg. 5, line 53-56 indicates 70 participants/35% twice.

- Again, related to the “Results” component of the Abstract, is there a way for the authors to summarize key findings across all major areas of inquiry as proposed in the “Objectives” and “Design” elements of the Abstract (i.e., prevalence of dementia and MCI, risk of externalized violence/self-harm, activities of daily living needs, mental health needs, brain injury, social networks, etc.)?

INTRODUCTION
- Pg. 7, line 10-14: In addition to recent research into dementia in prison emphasizing the need to identify prevalence and improve (specialized) services, research has also highlighted the importance of enhancing screening practices. That is, developing and/or tailoring screening tools for the prison context, given the challenges of assessing and diagnosing dementia in prison (and that screening tools have been developed for the community as opposed to prison context). For example, see: https://doi.org/10.1186/s40352-022-00168-8

- Pg. 7, line 20-22: When referencing patterns in other high-income countries, the following resource should also be included for the Canadian context as it is more recent (though it does only focus on people in federal custody): https://www.csc-scc.gc.ca/research/rib-19-03-en.shtml

- Pg. 7, line 27-45: In this paragraph the authors should clarify the country/context in which they are reporting findings, especially when noting prevalence rates for dementia (e.g., is it only England and Wales that is of focus in this paragraph?). Related to this point, while it is understandable to focus more attention on previous findings in the context of England and Wales, the authors should summarize findings from other countries when it comes to the issue of dementia and mild cognitive impairment among people in custody (particularly prevalence rates). This would provide a “bigger picture” vantage point of the issue. For example, see the following for a recent study in Canada that assessed the prevalence of dementia and community reintegration needs of a sample of older people in federal custody (which also summarizes prevalence rates from studies conducted in other countries): https://doi.org/10.1186/s40352-022-00168-8

- Pg. 8, line 12-14: For the first aim, it should be specified that the focus is on “older people in custody in England and Wales.”

- Pg. 8, line 16-17: With respect to the second aim, the authors should specify what is meant when referring to establishing “the degree and type of impairment.” In addition, the authors first note the focus on “risk of re-offending,” although previous discussion (including the Abstract) does not suggest this to be a focus (perhaps it is just slight variations in wording).

- Pg. 8, line 12-17: With respect to both the first and second aim, is the MoCA exclusively used to assess prevalence of MCI and as a screener for further assessment of dementia via the ACE-III? The key question here is: why not assess behavioural challenges, as well as health and social needs, according to both the ACE-III and MoCA (i.e., why is only the ACE-III of focus when conducting further analyses on behavioural, health, and social domains)?

METHODS
- Pg. 9, line 12-30: With respect to discussing the sampling, further detail could be provided regarding the prisons included in the sample. For instance, how many women's vs. men's prisons? What is the housing capacity or number of individuals housed in the prisons selected into the sample (at the time of the study)? It's also noted that one prison was selected as it possesses a dedicated wing for older and disabled individuals, though there are two such prisons in the country. What was the rationale for excluding the other prison with a dedicated wing for older and disabled individuals?

- Pg. 9, line 12-41: Related to the above point, further detail could be provided regarding the sampling frame/study setting. Again, what is the housing capacity or number of individuals in each of the selected prisons in total and, further, what is the total number of people in custody aged 50 and over in each of the prisons? In this case, it would be helpful to provide information on the population composition of the prisons (i.e., housing capacity or number of individuals housed at the time of the study) and an estimate of older people in custody in each of the prisons in general to better understand the composition of the prisons and where the sample was drawn from. A table might be most useful to organize this information. Please note that, in light of the importance of participant confidentiality, I'm only requesting general information on the prisons as opposed to any specific details.

- Pg. 9, line 12-30: While it is clear the number and types of prisons that were selected more generally, how exactly were the specific prisons selected? For instance, were prisons randomly selected and prison officials contacted about interest in the study? Were the prisons selected based on professional relationships between research team members and prison officials? Generally speaking, what procedure led to the exact prisons that were included in the sampling frame?

- Pg. 9, line 36-41: Given that the sample was stratified according to age and a specific proportion were sampled based on age range, what was the possible pool of participants across each age range? That is, how many men and women in custody in the sample prisons were 50-59, 60-69, 70-79, and 80+ prior to reaching the study sample?

- Generally speaking, the abovementioned points emphasize the need for greater

detail surrounding the study sample and setting and, more specifically, details of the study setting(s) that help contextualize what led to the final sample.

- Pg. 9, line 45: Title this subsection “Recruitment and Data Collection Procedures” as this is generally the focus of the section as it stands. Otherwise, separate recruitment and data collection procedures into two distinct sections. Also ensure that all aspects of data collection procedure are specified. Related to this point, it would be important to know the procedure for collecting data separately for the MoCA and ACE-III instruments and what this entailed. Some of the information is already provided, but more detail on how these instruments were administered would be beneficial. Additionally, were only two total interviews conducted? The first being the demographic and MoCA, and the second being more specific demographic (seemingly related to dementia assessment), the ACE-III and the additional assessment tools? Overall, a more fulsome and systematic description of the procedures on how individuals were recruited, and data were collected, would be important to better understand the steps the researchers took to collect data for all relevant constructs (and the tools/assessments/collection activities conducted along the way).

- Pg. 9, line 48: What is meant by the following sentence, “We estimated point prevalence according to a specific census date per site”?

- Pg. 10, line 16: What is used to assess degree and type of impairment? This is mentioned several times up to this point, but it’s not clear which tool is used to make such assessments.

- Pg. 10, line 32-59 & Pg. 11, line 3-8: With respect to the measures section, there are two key issues: (1) it reads primarily as a data collection procedure, and (2) the study variables, as well as the tools included in the study, are very briefly listed with little to no discussion on the characteristics of the tools. Furthermore, it is not clear whether the assessments for MCI or ACE-III are modified/tailored to suit the prison context (or whether they have been previously modified for this context). Discussion surrounding data collection procedures should be reserved for its own section and the measures section should provide a more fulsome description of the study variables and, most importantly, the tools that were used and their various characteristics. Also, it would be important to know why specific tools were selected for the study. For instance, what was the rationale behind using the MoCA and ACE-III for assessing cognitive impairment and dementia, respectively, versus other possible tools? What are the previous findings for reliability/validity of the tools used?

- Pg. 11, line 13-15: What is meant by the following line “The prevalence estimates were directly age-standardised using the age distribution of the national prison population as the reference”? Specifically, it is not clear what is meant by age-

standardization using the age distribution of the national prison population.

- Pg. 11, line 13-26: The data analytic procedure could be made clearer. Specifically, does the analysis focus exclusively on estimating prevalence estimates for dementia, mild cognitive impairment, and relevant behavioural issues, health needs, social needs, support networks, etc.? How were prevalence estimates calculated specifically for the MoCA and ACE-III? Are the produced prevalence estimates based on weighted estimates (and thus not sample estimates), or are both sample and weighted estimates calculated? Line 17-20 appears to suggest this, but it is not entirely clear.

- Pg. 11, line 21-25: Further to the above points related to data analytic procedure, producing age-specific prevalence estimates is fine in general; however, there is likely limited rationale for doing so on the basis of enabling comparison with community-based samples, especially as extant literature has argued that older people in prison experience ‘accelerated aging’ (i.e., experience age-related problems equivalent to those 10-15 years their elder in the community).

- With respect to data analytic procedure, the authors disaggregate according to age group to provide more granular findings but do not disaggregate according to gender for the same. Considering a key argument in the Introduction section was that studies have generally neglected to assess both older men and women in custody in terms of dementia/MCI, it would be crucial to include a gender-based analysis in this regard.

- Overall, the description of the data analysis should more closely align with the presentation of the Results section (or vice versa).

- Pg. 11, line 25-26: What was the exact extent of missing data across the study variables? Were missing data handled by case-wise deletion?

RESULTS
- Figure 1: The flow diagram shows that the ACE-III was completed for 74 individuals, however, it is not immediately apparent how this subsample came to be.

- Pg. 13, line 18-20: What was the % of participants who screened positively on the MoCA that had any history of a dementia diagnosis (or notes of cognitive dysfunction) in healthcare notes?

- Related to a point raised pertaining to the Methods section, the authors should provide prevalence estimates disaggregated by gender (though further disaggregation by age wouldn’t be necessary given the low base rate).

- Table 1: The authors should indicate that these results pertain specifically to the ACE-III. Relatedly, what about findings for MCI based on the MoCA presented in a similar way?

- Pg. 13, line 43-53: The authors present characteristics for those who screen positive on the ACE-III. Why not present characteristics based on those who screen positive on the MoCA as well?

- Pg. 14, line 3-43: With respect to the discussion on other conditions potentially related to MCI/dementia, are the authors exclusively reporting findings based on those who screened positive on the ACE-III? This should be specified. Also, why not examine based on those who screened positive on the MoCA as well?

DISCUSSION
- The first paragraph in the Discussion section (under “Main Findings”) can be removed as it is repetitive to what is already presented in the Results section.

- When comparing findings from previous studies, the authors should also extend discussion beyond the context of England and Wales (specifically for prison populations). Again, see the following for some discussion: https://doi.org/10.1186/s40352-022-00168-8

- In terms of limitations, the authors should note the limitation of screening (and diagnosing) dementia in prison contexts using instruments developed based in the community setting and, thus, the need to modify/tailor instruments (and further research this) for the prison setting.

- Overall, the Discussion section reads well and the discussion/interpretation of the findings are accurate and relevant to the data.

VERSION 1 - AUTHOR RESPONSE

Reviewer: 1

1. p. 7, line 6:
-Please spell out NHS.

Added page 5.

2. p. 8, line 24: "We aimed to recruit 860 prisoners (591 men and 269 women). This sample size was calculated to enable estimation of a prevalence value of 7%, based on a previously reported estimate (14), with …"

-Why limit your ability to estimate a higher prevalence? Please expand on why this decision was made and why you did not aim to recruit more prisoners in your sample.

Our statistician powered our study according to a previously reported prevalence estimate of 7%, in line with standard practice. We do not believe that we limited our ability to detect only a higher prevalence value as this was the best estimate that was available to us at the planning stage. Conducting 860 interviews was costly and time consuming and thus it was a significant burden to the prison service. Also, given the resources that were available to the research team, it would not have been feasible to recruit more prisoners and conduct a larger number of interviews.

3. pg. 8, line 55: "by the MCA…"
-Spell out MCA or add MCA in paratheses after Mental Capacity Act in the previous text.

MCA added on page 6.

4. p 10, line 13 and 14, "This was supplemented by routinely collected information gathered from SystmOne electronic health records (EHRs)….."
-Please provide more detail about the EHR's, the data they contain, and how they were used to supplement the information collected in the study. Was there a specific protocol in place or a standard set of variables that were pulled for each participant? What information was accessed? Who accessed this information? Were special permissions obtained? Clarification on this is needed.

The following text has been added to page 8.

SystmOne provides a single EHR for every patient. This shared record is available across all healthcare settings to any staff who need it during a patient's care. Participants consented to researchers accessing their EHR at each site. Researchers used a specifically designed proforma to collect information on past and current comorbidities and medications from the EHRs.

5. p 10, line 20-21, "Information pertaining to risks of self-harm and re-offending was sought from the Offender Management Unit….."
-Please clarify what self-harm and re-offending assessment information is included in the Offender Management Unit. When was this assessed? Is it reassessed periodically? Does it change during the individual's sentence?

The following information has been added on page 9:
We also sought the following information from the Offender Management Unit in each prison: 1) Offender Assessment System (OASys) rating of risk of harm to self and others (This incorporates static and dynamic risk and is categorised as very high, high, medium or low. It is reassessed at different time points depending on the individuals sentence type and prior to release). 2) Risk Matrix 2000 score (a risk assessment tool which uses static risk factors to predict the likelihood of reconviction for a sexual or violent offence).

6. p 10, line 22-26, "Consent was sought from the participant (or advice from the consultee) as regards researchers accessing prisoners EHRs, which were subsequently screened for any indication of diagnosed MCI or dementia."
-How many provided consent and how many did not? Could you have missed additional diagnoses?

Only 1 person did not consent to examination of EHR.

7. p 10, lines 33-36, "The MoCA was assessed between November 2016 and February 2018 …. Participants who scored less than 23 on the MoCA were invited to complete a further battery of assessments."
-Please clarify when further assessments were taken. It's not clear if they were performed Immediately after the MoCA or at another time. Also, when were the prisoner's EHR's accessed. A clear description of the timeline will be helpful.

Added to page 8:
This (second battery of assessments) was conducted as soon as possible after the initial assessment but not on the same day
Added page 9:
The information from the OMU and EHRs was collected after all assessments were complete.

8. p 11, line 29-32, "Patient and Public Involvement"
-Can this section be moved to the acknowledgements? It might fit better there.

Our preference is that the PPI section remains in the text to illustrate its importance but will move if required.

9. p 12, Figure 1,
- Is it possible to report a comparison of those included in the study and those excluded to assess any potential bias?

Unfortunately, we do not have access to this data.

10. p 13, line 18-20, "Only 2 individuals (3%) who screened positively on the ACE-III had a diagnosis of dementia in their prison healthcare notes, suggesting current under-detection. …."
-This doesn't agree with the conclusion on p16, line 22-24. Please review and clarify your take home message.

The 2% refers to what was recorded in EHR.
The 7% refer to the findings from our assessments.
I have added a clarifying statement to this effect in the conclusion on page 16 and on page 12.

11. p 15, line 12-24, "We compared the prevalence rates of dementia amongst the older prisoner population with those that have been reported for the wider community."
- Where did the "wider community" data come from? This information needs to be included in the methods section and ordered in line with how the results are presented.

We have moved the comparison paragraph to the findings on page 13 and changed the reference in the methods section to match the community comparison findings.

12. p 15, line 41-43, "Our result of 7.0% is higher than some previous results 1-7%."
- These results are lower than similar studies in the US. See Miller, et al., Dementia in the incarcerated population: a retrospective study using the South Carolina Alzheimer's disease registry, USA. DOI 10.1108/IJPH-08-2021-0071. Also, take a look at other prevalence studies in Europe including Fazel et al., 2001; Kingston et al., 2011; Combalbert et al., 2018.

Thank you for highlighting this point. We have changed the sentence on page 15 to…
Our result of 7.0% is higher than some previous results (14,31) and lower than others (2–4) with previous estimates ranging between 1 and 20% globally.

13. p 15, line 48, "…team (32), suggesting significant under-recognition of dementia/MCI in prisons."
- Also, potential missed dementia/MCI diagnosis prior to prison sentence.

Added "and potential prior to prison" to sentence p. 15.

14. p 15, line 51-56, "However, these differences may be actual and accounted for in part…."
- This paragraph has more to do with who was reporting the diagnosis and not why the prison population has high diagnosis. These lines would fit better on page 14, paragraph 1.

Moved as requested.

15. p 16, line 22-24, "Overall, it is likely that this led to an overestimation of the prevalence…"
- Need to cite some statistics comparing the number of diagnoses made by physicians and those made by non-physicians using validated screening tools only. This is needed to confirm your conclusion here.

This data is not available. We have therefore re-phrased on page 15 to "It's is possible that this led to an overestimation of the prevalence.."

16 p 16, line 41-48, "For the MoCA, we suggest a cut-off score of 23…"
- Please expand on the implications of this choice. Has the MoCA been validated in similar populations? Be sure to include information on the need for studies to validate the use of MoCA in the prison population.

The MoCA has not been validated in the prison population. It has been validated for other vulnerable populations, as described. We used the cut off for other vulnerable populations as this was the best available. The following sentence was added on page
"It would also be helpful to validate the MoCA for use in prison population in future studies."

17. p 16, line 58, "…sentencing for historical, mainly sexual offences."
- Is there any literature to support this? Please cite a reference(s) here.

Reference added.

18. p 17, line 9-15, "….outcomes of a sample of ACE-III-positive individuals over a period of 3–5 years"
- What about MoCA?

MoCA added page 16.

19. p 16, line 22-23, "In summary, we have found that 7% of the older prisoner population (1020 individuals) have dementia…"
- I suggest a revision like this to include all main findings:
"We found that the prevalence of dementia was 7.0% for ages 50+ and 11.8% in prisoners 70+. In addition, the majority of prisoners who screened positive on the ACE-III had a high or very high risk of harm to self or others and reported having no friends to call on for help."

Altered as suggested page 17.

Additional suggestions:
Please be sure all results are introduced in the discussion. I believe these will strengthen your paper and help its readability.

Added.

Reviewer: 2

1. - The authors should be cognizant of language throughout when it comes to reference of clinically significant symptoms of dementia (and cognitive impairment) vs. a dementia diagnosis. In this case, screening instruments are used and, thus, a formal diagnosis is not made. On pg. 13, line 36-38 the authors state, "It is estimated that 7% of these prisoners have dementia." However, it is most appropriate to suggest that 7% of people in custody have clinically significant symptoms of dementia (and/or mild cognitive impairment).

Thank you for highlighting this. Wording changed on page 12 to 'symptoms of dementia'.

2. ABSTRACT
- With respect to the "Setting" component of the Abstract, the authors should specify the n's for prisons included in the study (separately for men and women prisons).
This is now included in the abstract.
Men's (n=11)
Women's (n=10)

3. - With respect to the "Main Outcome Measure" component of the Abstract, is the MoCA not included as a main outcome?
The MoCA is not the main outcome.

4. - With respect to the "Results" component of the Abstract, a few minor points: (1) the authors should include prevalence estimates not only as a whole, but also for men and women separately regarding the ACE-III (dementia) and MoCA (MCI); (2) when referencing MCI it should be indicated in one way or another this is in reflection of MoCA screening results; and (3) findings for behavioural risk (i.e., harm to self/others) and needs/social networks are noted within the context of screening results for dementia (ACE-III), but what about mild cognitive impairment (MoCA)?

4.1. Thank you for highlighting this. The following statement has been added to the abstract and page 12. |"The prevalence of dementia for men was 7.0% (CI 5.2, 9.4) and for women was 6.0% (CI 3.8, 9.5).
4.2 For clarification the ACE III and the MoCA both examine MCI and dementia. The ACE III however does this in much more detail and includes cut off points for dementia and MCI. The MoCA does not include these cut off points.
4.3 Information on behavioural risk and social networks was only collected for individuals who screened in on the MoCA and therefore completed the ACE III. This is stated on page 8 "Participants who scored less than 23 on the MoCA were invited to complete a further battery of assessments." Every individual who competed the ACE III.

5. An additional minor point: Pg. 5, line 53-56 indicates 70 participants/35% twice.

Deleted – thank you.;

6. - Again, related to the "Results" component of the Abstract, is there a way for the authors to summarize key findings across all major areas of inquiry as proposed in the "Objectives" and "Design" elements of the Abstract (i.e., prevalence of dementia and MCI, risk of externalized violence/self-harm, activities of daily living needs, mental health needs, brain injury, social networks, etc.)?

We feel the current layout is satisfactory.

7. INTRODUCTION
- Pg. 7, line 10-14: In addition to recent research into dementia in prison emphasizing the need to identify prevalence and improve (specialized) services, research has also highlighted the importance of enhancing screening practices. That is, developing and/or tailoring screening tools for the prison context, given the challenges of assessing and diagnosing dementia in prison (and that screening tools have been developed for the community as opposed to prison context). For example, see: https://doi.org/10.1186/s40352-022-00168-8

Thank you – added page 6.

8. - Pg. 7, line 20-22: When referencing patterns in other high-income countries, the following resource should also be included for the Canadian context as it is more recent (though it does only focus on people in federal custody): https://www.csc-scc.gc.ca/research/rib-19-03-en.shtml

Sorry this link did not work.

9. - Pg. 7, line 27-45: In this paragraph the authors should clarify the country/context in which they are reporting findings, especially when noting prevalence rates for dementia (e.g., is it only England and Wales that is of focus in this paragraph?). Related to this point, while it is understandable to focus more attention on previous findings in the context of England and Wales, the authors should summarize findings from other countries when it comes to the issue of dementia and mild cognitive impairment among people in custody (particularly prevalence rates). This would provide a "bigger picture" vantage point of the issue. For example, see the following for a recent study in Canada that assessed the prevalence of dementia and community reintegration needs of a sample of older people in federal custody (which also summarizes prevalence rates from studies conducted in other countries): https://doi.org/10.1186/s40352-022-00168-8

Added information about global prevalence rates.

10. - Pg. 8, line 12-14: For the first aim, it should be specified that the focus is on "older people in custody in England and Wales."
The word 'older' has been added – thank you.

11. - Pg. 8, line 16-17: With respect to the second aim, the authors should specify what is meant when referring to establishing "the degree and type of impairment." In addition, the authors first note the focus on "risk of re-offending," although previous discussion (including the Abstract) does not suggest this to be a focus (perhaps it is just slight variations in wording).

The second aim has been reworded to:

> *2. Establish the risk of harm to self and others, Activities of Daily Living needs and social networks of those who screen positive on the ACE-III.*

- Pg. 8, line 12-17: With respect to both the first and second aim, is the MoCA exclusively used to assess prevalence of MCI and as a screener for further assessment of dementia via the ACE-III? The key question here is: why not assess behavioural challenges, as well as health and social needs, according to both the ACE-III and MoCA (i.e., why is only the ACE-III of focus when conducting further analyses on behavioural, health, and social domains)?

Further assessments were conducted on everyone who screened positively on the MoCA. This is stated on page 8. Apologies if this is not clear.

METHODS

- Pg. 9, line 12-30: With respect to discussing the sampling, further detail could be provided regarding the prisons included in the sample. For instance, how many women's vs. men's prisons? What is the housing capacity or number of individuals housed in the prisons selected into the sample (at the time of the study)? It's also noted that one prison was selected as it possesses a dedicated wing for older and disabled individuals, though there are two such prisons in the country. What was the rationale for excluding the other prison with a dedicated wing for older and disabled individuals?

Paragraph below added

The sample was drawn randomly from all women's prisons (n=10) (with the exception of one Women's prison that refused to participate) and a representative range of adult male prisons (n = 11) across England and Wales, including local prisons holding individuals on remand, serving short sentences and in the early part of long sentences; training and dispersal prisons holding men part-way through long sentences; high-secure establishments holding those considered to be high risk; and open prisons holding short-term prisoners deemed low risk and those in the final stages of long sentences in preparation for community release. We selected sites based on the proportion of each prison type in the prison estate as a whole- 1 of the 8 high secure sites; 3 of the 31 local prisons; 5 of 51 category B or C training and dispersal prisons and 1 of the 7 category D/open prisons (as defined by the justice.gov.uk prison index). We selected the prisons from across England to include a broad geographical spread and for pragmatic reasons. This included one prison in the North East, three in the North West, two in Yorkshire, one in the midlands and two in London. We also included a prison that has a specific wing for older and disabled prisoners. This was one of only two prisons in the country with a dedicated wing for prisoners based on their age and health or social care needs. One of these two prisons was selected for pragmatic reasons.

- Pg. 9, line 12-41: Related to the above point, further detail could be provided regarding the sampling frame/study setting. Again, what is the housing capacity or number of individuals in each of the selected prisons in total and, further, what is the total number of people in custody aged 50 and over in each of the prisons? In this case, it would be helpful to provide information on the population composition of the prisons (i.e., housing capacity or number of individuals housed at the time of the study) and an estimate of older people in custody in each of the prisons in general to better understand the composition of the prisons and where the sample was drawn from. A table might be most useful to organize this information. Please note that, in light of the importance of participant confidentiality, I'm only requesting general information on the prisons as opposed to any specific details.

This information is unavailable.

- Pg. 9, line 12-30: While it is clear the number and types of prisons that were selected more generally, how exactly were the specific prisons selected? For instance, were prisons randomly selected and prison officials contacted about interest in the study? Were the prisons selected based on professional relationships between research team members and prison officials? Generally speaking, what procedure led to the exact prisons that were included in the sampling frame?

The precise prisons were pragmatically selected. Prison governors were approached for the specific prisons that had been selected. - line altered accordiingly.

- Pg. 9, line 36-41: Given that the sample was stratified according to age and a specific proportion were sampled based on age range, what was the possible pool of participants across each age range? That is, how many men and women in custody in the sample prisons were 50-59, 60-69, 70-79, and 80+ prior to reaching the study sample?

Table 1 below has been added in line with sampling strategy.

Number of men and women in prison in England and Wales by age

Age group	All	Men	Women
50-59	8638	8227	411
60-69	3243	3149	94
70+	1641	1624	17
Total	84373	80454	3919

- Generally speaking, the abovementioned points emphasize the need for greater detail surrounding the study sample and setting and, more specifically, details of the study setting(s) that help contextualize what led to the final sample.

Details above

- Pg. 9, line 45: Title this subsection "Recruitment and Data Collection Procedures" as this is generally the focus of the section as it stands. Otherwise, separate recruitment and data collection procedures into two distinct sections. Also ensure that all aspects of data collection procedure are specified. Related to this point, it would be important to know the procedure for collecting data separately for the MoCA and ACE-III instruments and what this entailed. Some of the information is already provided, but more detail on how these instruments were administered would be beneficial. Additionally, were only two total interviews conducted? The first being the demographic and MoCA, and the second being more specific demographic (seemingly related to dementia assessment), the ACE-III and the additional assessment tools? Overall, a more fulsome and systematic description of the procedures on how individuals were recruited, and data were collected, would be important to better understand the steps the researchers took to collect data for all relevant constructs (and the tools/assessments/collection activities conducted along the way).

Title changed and further detail added with track changes.

- Pg. 9, line 48: What is meant by the following sentence, "We estimated point prevalence according to a specific census date per site"?

"According to a specific census date for each prison site, we estimated point prevalence (with disease cases observed on a given single day) rather than point prevalence (with cases ascertained across a one week, one month or one year period)".

- Pg. 10, line 16: What is used to assess degree and type of impairment? This is mentioned several times up to this point, but it's not clear which tool is used to make such assessments.

The ACE-III measured 'degree and type of impairment' but reference to this statement has been removed to avoid confusion.

- Pg. 10, line 32-59 & Pg. 11, line 3-8: With respect to the measures section, there are two key issues: (1) it reads primarily as a data collection procedure, and (2) the study variables, as well as the tools included in the study, are very briefly listed with little to no discussion on the characteristics of the tools. Furthermore, it is not clear whether the assessments for MCI or ACE-III are modified/tailored to suit the prison context (or whether they have been previously modified for this context). Discussion surrounding data collection procedures should be reserved for its own section and the measures section should provide a more fulsome description of the study variables and, most importantly, the tools that were used and their various characteristics. Also, it would be important to know why specific tools were selected for the study. For

instance, what was the rationale behind using the MoCA and ACE-III for assessing cognitive impairment and dementia, respectively, versus other possible tools? What are the previous findings for reliability/validity of the tools used?

The statement – "These tools have previously been used in prison based studies (5,6) but have not been specifically validated for the prison population." – has been added.

For clarification, both the MOCA and the ACE assess for MCI and dementia. The ACE is however more detailed.
The strengths and limitations of the tools are discussed in the 'strengths and limitations' section.
There is not sufficient word space to describe each of the tools in detail, however references are provided for further information.

- Pg. 11, line 13-15: What is meant by the following line "The prevalence estimates were directly age-standardised using the age distribution of the national prison population as the reference"? Specifically, it is not clear what is meant by age-standardization using the age distribution of the national prison population.

In doing so we multiplied the age-specific prevalence values that we observed in the study population by the number of persons in each equivalent age group in the national (reference or 'standard') population. This generated the expected number of prevalent cases for each age group in the study population, which were then summed across all age groups. Finally, to calculate the age-adjusted prevalence value, we divided the total number of expected deaths (the numerator) by the whole standard population (the denominator).

- Pg. 11, line 13-26: The data analytic procedure could be made clearer. Specifically, does the analysis focus exclusively on estimating prevalence estimates for dementia, mild cognitive impairment, and relevant behavioural issues, health needs, social needs, support networks, etc.?

As is reported in the Results section of the abstract and the main text of the paper, we reported prevalence estimates for dementia and mild cognitive impairment. We did not estimate or report prevalence estimates for behavioural issues, health needs, social needs, support networks, etc.

How were prevalence estimates calculated specifically for the MoCA and ACE-III?

We did not estimate prevalence specific to the MoCA and the ACE-III. As is explained in the 'Measures' subsection of the Methods, participants who scored less than 23 on the MoCA were invited to complete a further battery of measures that included the ACE-III. With this sequential multi-phase approach, it was not possible to estimate prevalence values separately for the MoCA and for the ACE-III, and this was not one of the study's stated aims anyway.

Are the produced prevalence estimates based on weighted estimates (and thus not sample estimates), or are both sample and weighted estimates calculated? Line 17-20 appears to suggest this, but it is not entirely clear.

As is explained in the ‘Data analysis’ subsection of the Methods, we applied probability weights to all prevalence estimates to ensure that the reported values were applicable to prison populations from which the study’s sample was drawn. Reporting estimates without first weighting them in this fashion would be uninformative.

- Pg. 11, line 21-25: Further to the above points related to data analytic procedure, producing age-specific prevalence estimates is fine in general; however, there is likely limited rationale for doing so on the basis of enabling comparison with community-based samples, especially as extant literature has argued that older people in prison experience ‘accelerated aging’ (i.e., experience age-related problems equivalent to those 10-15 years their elder in the community).

We do not understand the reviewer’s comment. Whilst we agree with the point about accelerated aging in prison populations, by generating age-specific prevalence estimates we have optimised the degree to which we can make comparisons between what was observed in our study and what has previously been reported in the literature.

- With respect to data analytic procedure, the authors disaggregate according to age group to provide more granular findings but do not disaggregate according to gender for the same. Considering a key argument in the Introduction section was that studies have generally neglected to assess both older men and women in custody in terms of dementia/MCI, it would be crucial to include a gender-based analysis in this regard.
Now stated on Page 13 - The prevalence of dementia for men was 7.0% (CI 5.2, 9.4) and for women was 6.0% (CI 3.8, 9.5).

- Overall, the description of the data analysis should more closely align with the presentation of the Results section (or vice versa).

The changes detailed above should now make this clearer.

RESULTS
- Figure 1: The flow diagram shows that the ACE-III was completed for 74 individuals, however, it is not immediately apparent how this subsample came to be.
All individuals who screened in on the MoCA complete the ACE III. A statement has been added to the figure.

- Pg. 13, line 18-20: What was the % of participants who screened positively on

the MoCA that had any history of a dementia diagnosis (or notes of cognitive dysfunction) in healthcare notes?

Only 2 individuals (3%) who screened positively on the MoCA had a diagnosis of dementia in their prison healthcare notes, this suggests current under-detection when compared to the findings of our assessments.

Related to a point raised pertaining to the Methods section, the authors should provide prevalence estimates disaggregated by gender (though further disaggregation by age wouldn't be necessary given the low base rate).
This statement has been added page 13
The prevalence of dementia for men was 7.0% (CI 5.2, 9.4) and for women was 6.0% (CI 3.8, 9.5).

- Table 1: The authors should indicate that these results pertain specifically to the ACE-III. Relatedly, what about findings for MCI based on the MoCA presented in a similar way?

There were only 4 individuals who screened in on the MoCA who did not screen in on the ACE -III.

- Pg. 13, line 43-53: The authors present characteristics for those who screen positive on the ACE-III. Why not present characteristics based on those who screen positive on the MoCA as well?

There were only 4 individuals who screened in on the MoCA who did not screen in on the ACE -III.

- Pg. 14, line 3-43: With respect to the discussion on other conditions potentially related to MCI/dementia, are the authors exclusively reporting findings based on those who screened positive on the ACE-III? This should be specified. Also, why not examine based on those who screened positive on the MoCA as well.

The findings reflect all those who screened in on the MoCA as detailed in our methods section.

DISCUSSION
- The first paragraph in the Discussion section (under "Main Findings") can be removed as it is repetitive to what is already presented in the Results section.

Reviewer 1 asked for further information to be added to this paragraph so we have kept it in.

- When comparing findings from previous studies, the authors should also extend discussion beyond the context of England and Wales (specifically for prison populations). Again, see the following for some discussion: https://doi.org/10.1186/s40352-022-00168-8

Added

- In terms of limitations, the authors should note the limitation of screening (and diagnosing) dementia in prison contexts using instruments developed based in the community setting and, thus, the need to modify/tailor instruments (and further research this) for the prison setting.

- Overall, the Discussion section reads well and the discussion/interpretation of the findings are accurate and relevant to the data.

Reviewer: 2
Competing interests of Reviewer: None.

VERSION 2 - REVIEW

**Reviewer**	**2**
**Name**	**Stoliker, Bryce E.**
**Affiliation**	**University of Saskatchewan, Centre for Forensic Behavioural Science and Justice Studies**
**Date**	**26-Nov-2024**
**COI**	

The authors have sufficiently addressed the comments from the previous review.
